# Multisystemic *Streptococcus gallinaceus* infection in a six-banded armadillo from a zoo collection

**DOI:** 10.1177/10406387261470345

**Published:** 2026-08-12

**Authors:** Raúl A. Resendiz-Pozos, Cassandra Lapham-Simpson, Tessa E. LeCuyer, Maria Soper, Melissa Macías-Rioseco, Jennine Ochoa, Hernando D. Acevedo, Todd Cornish

**Affiliations:** Tulare Branch, School of Veterinary Medicine, University of California–Davis, Davis, CA, USA; Fresno Chaffee Zoo, Fresno, CA, USA; California Animal Health and Food Safety Laboratory System, and Department of Pathology, Microbiology, and Immunology, School of Veterinary Medicine, University of California–Davis, Davis, CA, USA; Tulare Branch, School of Veterinary Medicine, University of California–Davis, Davis, CA, USA; Tulare Branch, School of Veterinary Medicine, University of California–Davis, Davis, CA, USA; Tulare Branch, School of Veterinary Medicine, University of California–Davis, Davis, CA, USA; Tulare Branch, School of Veterinary Medicine, University of California–Davis, Davis, CA, USA; Tulare Branch, School of Veterinary Medicine, University of California–Davis, Davis, CA, USA

**Keywords:** meningoencephalitis, septicemia, six-banded armadillo, streptococcosis, zoonotic

## Abstract

A six-banded armadillo (*Euphractus sexcinctus*) from a zoo collection in central California developed acute neurologic signs followed by death. Postmortem examination revealed multisystemic bacterial disease (fibrinosuppurative meningoencephalomyelitis, splenitis, and endocarditis) with fibrinous necrotizing vasculitis and thrombosis. *Streptococcus gallinaceus* was isolated from the heart, lung, and meninges. To our knowledge, this microorganism has not been reported previously in association with systemic disease in an armadillo. Although the source of infection remains unknown, the animal was housed in the same building as chickens, a species that historically is associated with outbreaks of this bacterial infection. This case further broadens our understanding of the possible host range of *S. gallinaceus* and emphasizes the risk of interspecies pathogen transmission in captive environments.

*Streptococcus gallinaceus* is a gram-positive, catalase-negative bacterium that was first isolated from broiler chickens in 2002,^5^ with subsequent reports of disease cases in poultry and swine.^12^ In addition to septicemia, endocarditis and infarcts in multiple organs have been reported.^13^ The route of transmission has not been fully elucidated and, although outbreaks of *S. gallinaceus* septicemia have been described in chickens, horizontal transmission between co-housed chickens in an experimental infection model has not been reported.^2,5^ The bacterium is zoonotic, with human cases mainly reported in people with exposure to chickens. However, other poultry, as well as sheep, cattle, and goat carcasses, may also be sources of exposure.^1,13^ Clinical manifestations in humans include bacteremia, endocarditis, and osteomyelitis.^1,2,13^ *S. gallinaceus* has also been reported in red pandas (*Ailurus fulgens*) and American black bears (*Ursus americanus*) with pneumonia.^10,15^ A search on Google, PubMed, CAB Direct, Web of Science, and Scopus using the terms “*Streptococcus gallinaceus*” and “six-banded armadillo (*Euphractus sexcinctus*)” did not retrieve any case reports, suggesting that this condition has not been reported previously in armadillos. Here, we describe a case of multisystemic *S. gallinaceus* infection in a six-banded armadillo.

A 10-mo-old female six-banded armadillo (*Euphractus sexcinctus*) was discovered early in the morning in her enclosure, positioned upside down, cold to the touch, and quivering. The armadillo was immediately taken to the on-site veterinary hospital, where she was found to be severely hypothermic, bradycardic, tachypneic, and unresponsive to external stimuli. After broad supportive therapy, including oxygen supplementation, heat therapy, and IV fluids, the body temperature and heart and respiratory rates gradually normalized over several hours. Later that afternoon, the patient acutely decompensated, with bradycardia and bradypnea. Despite emergency supportive treatment and resuscitation attempts, the animal died. The armadillo was submitted to the California Animal Health and Food Safety Laboratory System (CAHFS; Tulare, CA, USA) for postmortem examination.

At autopsy, the carcass was in good postmortem condition, good body condition, with adequate adipose stores and normal muscle mass. Gross examination revealed abundant yellow, cloudy exudate, mixed with hemorrhage, that diffusely expanded the leptomeninges and extended into the adjacent neuroparenchyma and ventricles (**Fig. 1A**). No significant gross lesions were noted in the other examined organs. Tissues were fixed in 10% neutral-buffered formalin for 24 h, processed routinely to produce 4-μm sections, and stained with H&E and Gram Brown–Hopps stains.

**Figure 1.**
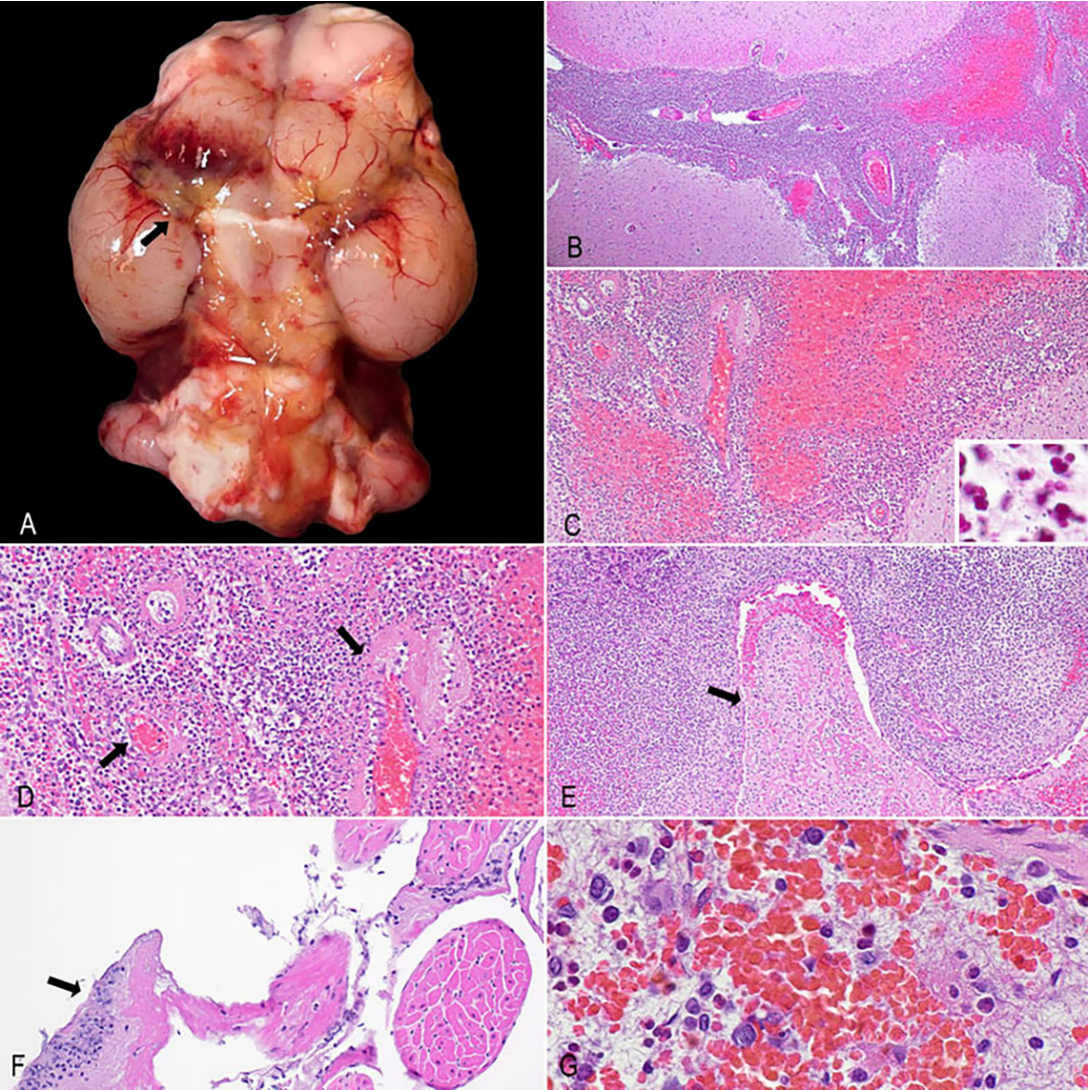
Gross and microscopic findings in a six-banded armadillo with systemic *Streptococcus gallinaceus* septicemia. **A.** Meninges of the ventrum of the brain expanded by abundant yellow exudate mixed with blood and fibrin (arrow). **B.** Leptomeninges markedly expanded by numerous inflammatory cells. H&E. **C.** The leptomeningeal infiltrate mixed with abundant blood and expanding into adjacent neuropil. H&E. Inset: gram-positive coccus, singly and in pairs. Gram stain. **D.** The leptomeningeal infiltrate with abundant neutrophils, macrophages, and a few lymphocytes. Fibrinoid necrosis of large- and medium-caliber arteries, with neutrophils infiltrating the intima and media (arrows). H&E. **E**. Meningeal vessel occluded by a fibrin thrombus (arrow) mixed with numerous neutrophils. H&E. **F.** Endocardium infiltrated by a few neutrophils mixed with fibrin (arrow). H&E. **G.** Splenic red pulp infiltrated by numerous neutrophils and histiocytes, mixed with fibrin. H&E.

Histologically, the leptomeninges of the brain and spinal cord were expanded by abundant infiltrating neutrophils, fewer macrophages and lymphocytes, fibrin, and intralesional gram-positive cocci (occurring singly or in pairs; diplococci). In some areas of the brain, foci of inflammation were present in the neuropil and ventricles. Adjacent neurons had variable degrees of degeneration and necrosis with associated gliosis. Vessels within the leptomeninges had fibrinous necrosis and infiltration of neutrophils in the intima and media, and some were occluded by fibrin thrombi. The spleen and endocardium had similar inflammatory changes (**Fig. 1B–G**).

Aerobic bacterial cultures were performed on brain, lung, and heart. Representative swabs were collected and inoculated onto 5% sheep blood agar (Hardy) and MacConkey agar (Hardy), streaked for isolation, and incubated at 35 ± 2°C with 5–10% CO_2_ for 24–48 h. The predominant bacterial growth in all 3 specimens was identified as *S. gallinaceus*. Additional bacteria were isolated from the heart (*Escherichia coli* and *Enterobacter cloacae* complex) and the lung (*E. coli*) specimens in lower numbers. The overall interpretation of the cultures was that *S. gallinaceus* was the organism most likely to be clinically significant, although a contribution from *E. coli* cannot be ruled out based on its presence in 2 of the 3 tissues cultured. Bacterial isolates were identified via matrix-assisted laser desorption/ionization time-of-flight mass spectrometry (Microflex LT/SH Biotyper; Bruker) with all scores ≥2.1. In addition, biochemical testing (API 20 STREP; bioMérieux) was consistent with reactions that have been described for *S. gallinaceus*.^2^

In vitro antimicrobial susceptibility testing was performed on *S. gallinaceus* isolated from the brain at the UC Davis Clinical Diagnostic Laboratories Microbiology Laboratory (Davis, CA, USA). Minimum inhibitory concentrations of antimicrobials were determined by broth microdilution in Mueller–Hinton broth with lysed horse blood on a custom Sensititre panel (CMV6SUCD; ThermoFisher). No clinical breakpoints exist for armadillos; hence, low-confidence interpretations were based on extrapolated data from other host species, using equine *Streptococcus* and human viridans *Streptococcus* spp. breakpoints (CLSI VET01S,^3^ CLSI M100^4^). Like many viridans streptococci, the isolate was susceptible to penicillin (MIC ≤0.06 µg/mL).^14^

*S. gallinaceus* was considered the primary septicemic pathogen, causing neutrophilic and fibrinous meningoencephalomyelitis, splenitis, and endocarditis, with fibrinous necrotizing vasculitis and thrombosis. *S. gallinaceus* is an emerging threat to the poultry industry.^2,14^ This organism has been linked to both human and wildlife infections, producing pneumonia in red pandas and black bears.^10^ The disease associated with this bacterium has not been reported previously in armadillos, and additional species may be susceptible. Viridans group *Streptococcus* spp., which include *S. gallinaceus*, are normal mucous membrane microbiota in a variety of host species,^7,11^ but it is unknown whether this organism can be found as a commensal in armadillos. Factors such as immune compromise and environmental stress could have contributed to this animal’s susceptibility to infection. Captivity can act as a potent chronic stressor that, although potentially mitigable, may predispose some exotic animal species to opportunistic infections.^8,9^

Cross-species transmission and the emergence of zoonotic diseases occur at complex animal–human–environmental interfaces within dynamic social-ecological systems shaped by human behavior, demographic shifts, and global changes.^6^ In our case, the source of exposure to *S. gallinaceus* is unknown. The armadillo was part of the zoo ambassador program and was housed in a building with various other animal species, including 2 chickens. The distance between the chickens and the armadillo was ~1.8 m. Animals in the ambassador program are periodically moved around the zoo grounds and off-site for public education purposes. The armadillo did not share an enclosure with other animals, and no poultry products were used in her diet or in her enclosure. Also, no comorbidities were reported before the death. The animal’s proximity to chickens and its close contact with zookeepers who move throughout various animal spaces are potential sources of exposure via cross-contamination and/or fomite transmission.
